# Supplementary material for: The Emulsification and Stabilization Mechanism of an Oil-in-Water Emulsion Constructed from Tremella Polysaccharide and Citrus Pectin
Source: Foods. 2024 May 16;13(10):1545. doi: 10.3390/foods13101545 (PMC11120492; doi:10.3390/foods13101545)
Supplement: Supplementary file 1 [file foods-13-01545-s001.zip › foods-2980332-supplementary.pdf]

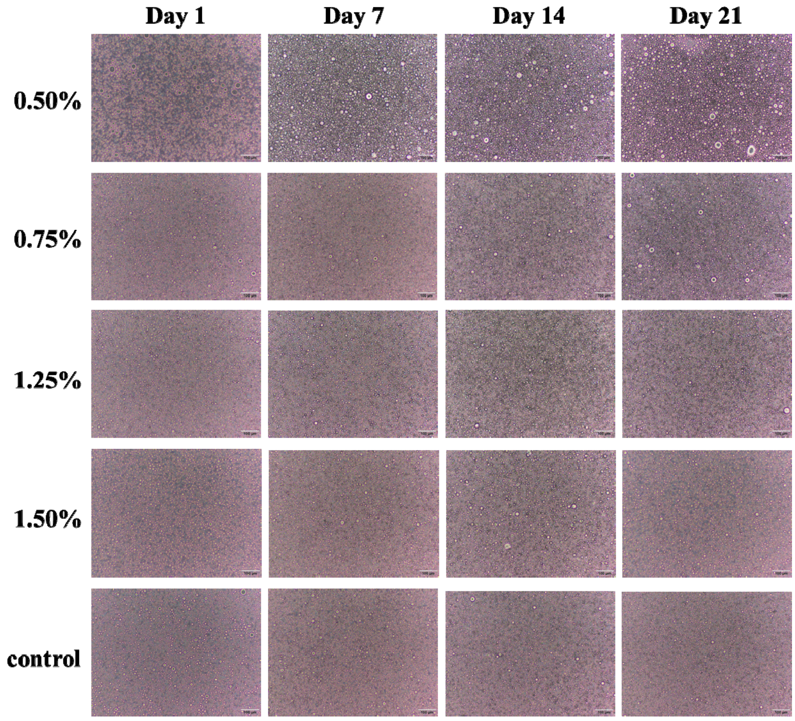

Figure S1. The morphologies of emulsion systems stabilized by TP:CP=5:5 under different polysaccharide concentrations (0.5%, 0.75%, 1.25%, 1.5%), with the emulsion stabilized by 1.0% TP:CP=5:5 as control.

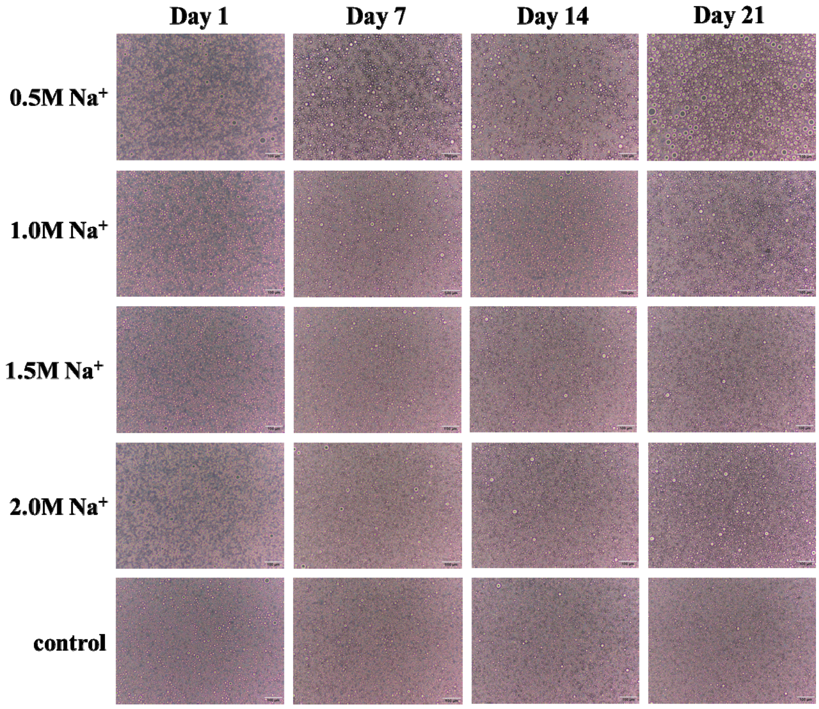

Figure S2. The morphologies of emulsion systems stabilized by 1.0% TP:CP=5:5 at different Na^+ strength (0.5 M, 1.0 M, 1.5 M, 2.0 M), with the emulsion stabilized by 1.0% TP:CP=5:5 as control.

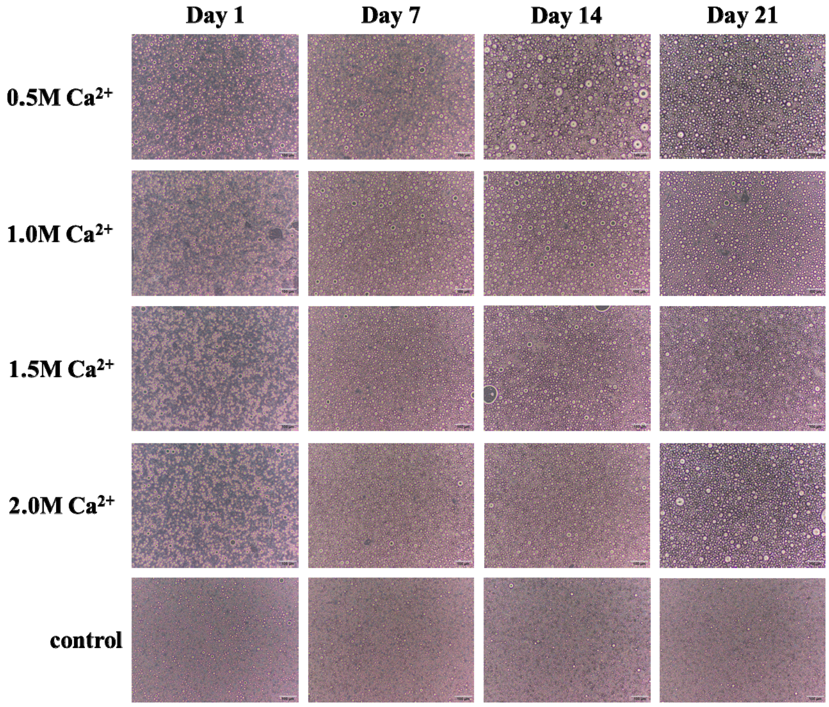

Figure S3. The morphologies of emulsion systems stabilized by 1.0% TP:CP=5:5 at different Ca^{2+} strength (0.5 M, 1.0 M, 1.5 M, 2.0 M), with the emulsion stabilized by 1.0% TP:CP=5:5 as control.

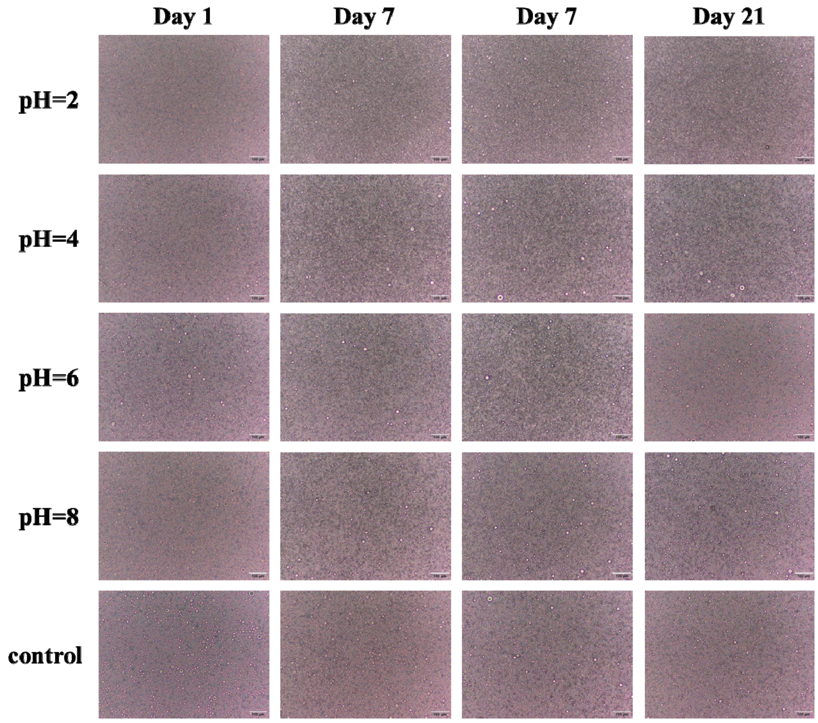

Figure S4. The morphologies of emulsion systems stabilized by 1.0% TP:CP=5:5 at different pH levels (pH=2, 4, 6, 8), with the emulsion stabilized by 1.0% TP:CP=5:5 as control.

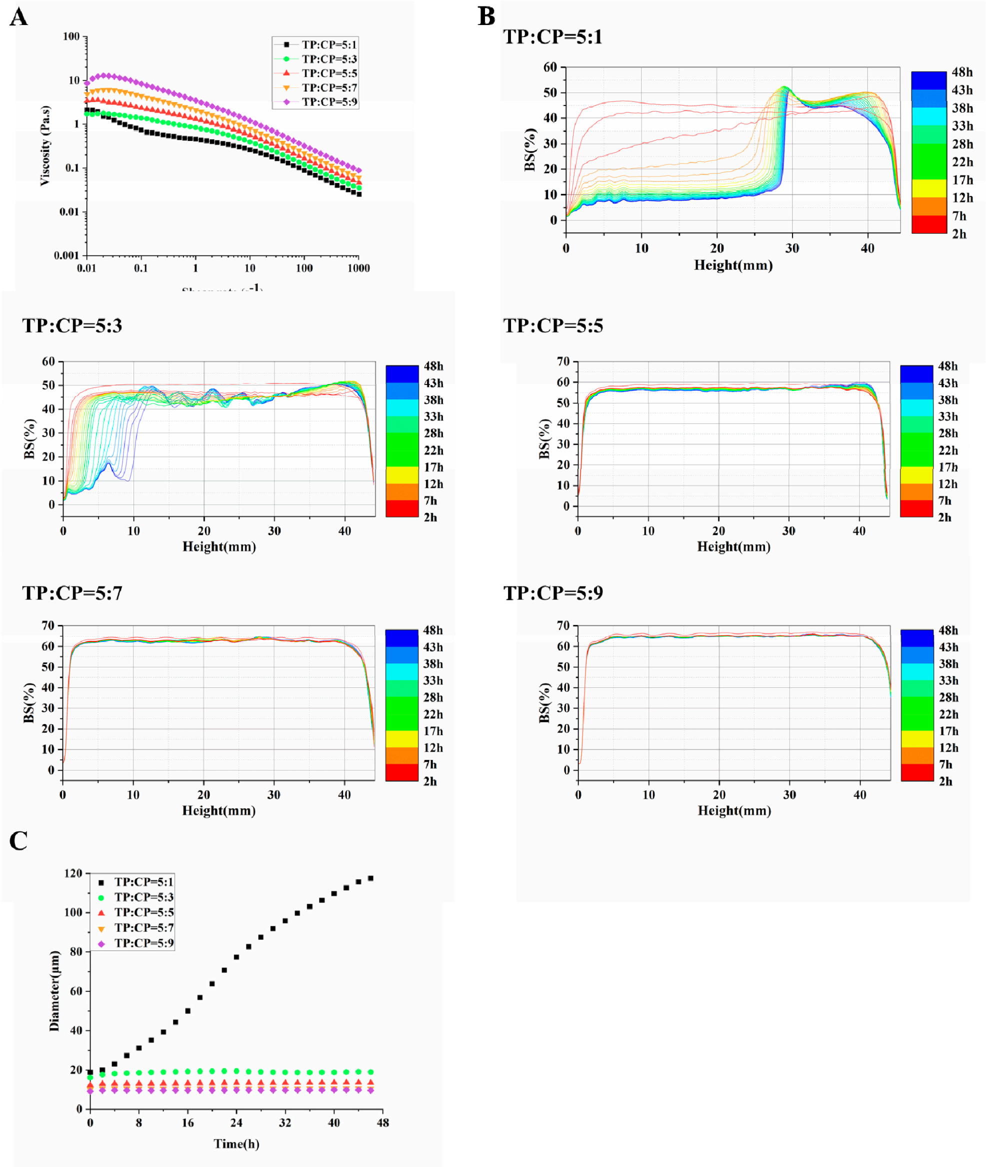

Figure S5. (A) Rheological properties of emulsion systems stabilized by TP-CP at different proportion (the concentration of TP in aqueous phase was 0.5%), (B) backscattering profiles of different emulsion systems during 2 days at 37 °C for accelerating emulsification instability, (C) the average droplets size of different emulsion systems.

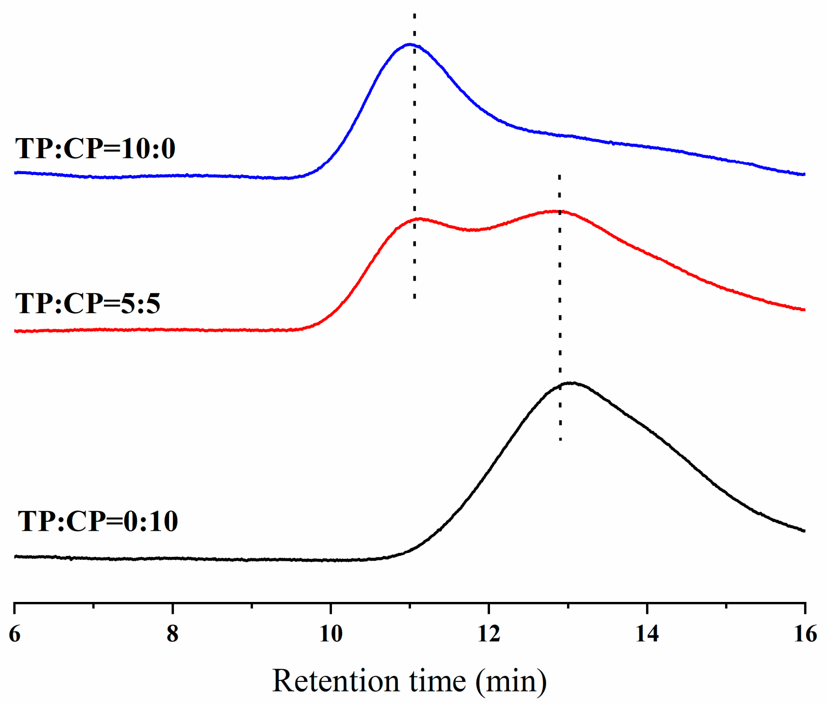

Figure S6. The molecular weight distribution of TP, CP and their mixture (TP:CP=5:5).
